# Molecular Architecture of  Yeast Chromatin Assembly Factor 1

**DOI:** 10.1038/srep26702

**Published:** 2016-05-25

**Authors:** Daegeun Kim, Dheva Setiaputra, Taeyang Jung, Jaehee Chung, Alexander Leitner, Jungmin Yoon, Ruedi Aebersold, Hans Hebert, Calvin K. Yip, Ji-Joon Song

**Affiliations:** 1 Department of Biological Sciences, KAIST Institute for the BioCentury, Cancer Metastasis Control Center, KAIST, 291 Daehakro Yuseong Daejeon, 34141, Korea; 2 Department of Biochemistry and Molecular Biology, The University of British Columbia, Vancouver, British Columbia, V6T 1Z3, Canada; 3 Department of Biosciences and Nutrition, Karolinska Institute, and School of Technology and Health, KTH Royal Institute of Technology, Novum, SE-141 57, Sweden; 4 Department of Biology, Institute of Molecular Systems Biology, ETH Zurich, 8093 Zurich, Switzerland; 5 Faculty of Science, University of Zurich, Zurich, Switzerland

## Abstract

Chromatin Assembly Complex 1 (CAF-1) is a major histone chaperone involved in deposition of histone H3 and H4 into nucleosome. CAF-1 is composed of three subunits; p150, p60 and p48 for human and Cac1, Cac2 and Cac3 for yeast. Despite of its central role in chromatin formation, structural features of the full CAF-1 in complex with histones and other chaperones have not been well characterized. Here, we dissect molecular architecture of yeast CAF-1 (yCAF-1) by cross-linking mass spectrometry (XL-MS) and negative stain single-particle electron microscopy (EM). Our work revealed that Cac1, the largest subunit of yCAF-1, might serve as a major histone binding platform linking Cac2 and Cac3. In addition, EM analysis showed that yCAF-1 adopts a bilobal shape and Cac1 connecting Cac2 and Cac3 to generate a platform for binding histones. This study provides the first structural glimpse of the full CAF-1 complex and a structural framework to understand histone chaperoning processes.

Chromatin is the high order structure of eukaryotic DNA and is composed of a repeating unit known as the nucleosome. Nucleosome is a complicated structure composed of 146 base pairs of DNA and dimers of histone H3, H4, H2A and H2B. The nucleosome is the key target of chromatin modification by several mechanisms. Many covalent modifications including methylation, acetylation, phosphorylation, crotonylation as well as ubiquitylation decorate histone tails, and different types and locations of these tail modifications result in different outcomes in terms of gene expression. Nucleosome is also remodeled by ATP-dependent chromatin remodelers to alter the composition and structure of nucleosome, and in turn regulate gene expression. Furthermore, dynamic assembly and disassembly of nucleosome itself is tightly regulated with various cellular processes such as DNA replication, DNA damage repair and transcriptions^1^.

The assembly of nucleosome occurs in step-wise manner and is mediated by various histone chaperones and enzymes. Assembly of the histone H3/H4 dimer is mediated by Heat shock protein 90 (HSP90), Nuclear Auto-antigen Sperm Protein (NASP), Hat1-RbAp48 histone acetyltransferase and Anti-Silencing Factor-1 (Asf1) in the cytosol. Histone H3/H4 dimer in complex with Asf1 is then imported into nucleus via Importin-4. In the nucleus, CAF-1 is responsible for depositing histone H3/H4 onto DNA during DNA replication and DNA repair, while HIRA deposits histone H3/H4 on DNA during DNA synthesis-independent nucleosome assembly. For histone H2A/H2B deposition, different assembly machineries including FACT and NAP1 are involved.

CAF-1 is the major histone chaperone complex for histone H3/H4 and composed of three subunits: p150, p60 and p48 for human, Cac1, Cac2 and Cac3 for yeast, and p180, p105 and p55 for fly^2^. Human p150 subunit has been shown to interact with histone H3/H4 and the p60 subunit, while fly p180 is thought to bridge p105 and p55^3^,^4^,^5^. Crystal structures of human RbAp48 (p48) and fly p55 in complex with the first helix of Histone H4, which is buried in H3/H4 dimer structure, indicate that p48 and p55 binding induces substantial structural changes in histones^6^,^7^. CAF-1 also binds to non-histone proteins. The N-terminal region of human p150 has been shown to interact with PCNA implicated in DNA repair pathway^8^. Human p60 and yeast Cac2 were found to bind to another histone chaperone Asf1 and this interaction is also mediated by histone H3/H4^9^,^10^,^11^,^12^. CAF-1 receives histone H3/H4 from other histone chaperones. It has been shown that the binding of p48 in CAF-1 to histone H3/H4 weakens the binding of Asf1 toward histone H3/H4, suggesting that there is an allosteric exchange of histone H3/H4 between Asf1 and RbAp48^13^.

CAF-1 is identified as a major histone chaperone involved in DNA replication and DNA repair pathways several decades ago. Despite many biochemical and structural studies on its subunits with histone and other binding partners, the structural feature of CAF-1 as a whole complex has not been characterized. Here, we characterize the molecular architecture of the yeast yCAF-1 complex by combining chemical cross-linking mass spectrometry (XL-MS) and single-particle EM.

## Results and Discussion

### Dissecting Molecular Interactions among CAF-1 complex, histones, and Asf1 by crosslinking mass spectrometry

**Figure 1:**
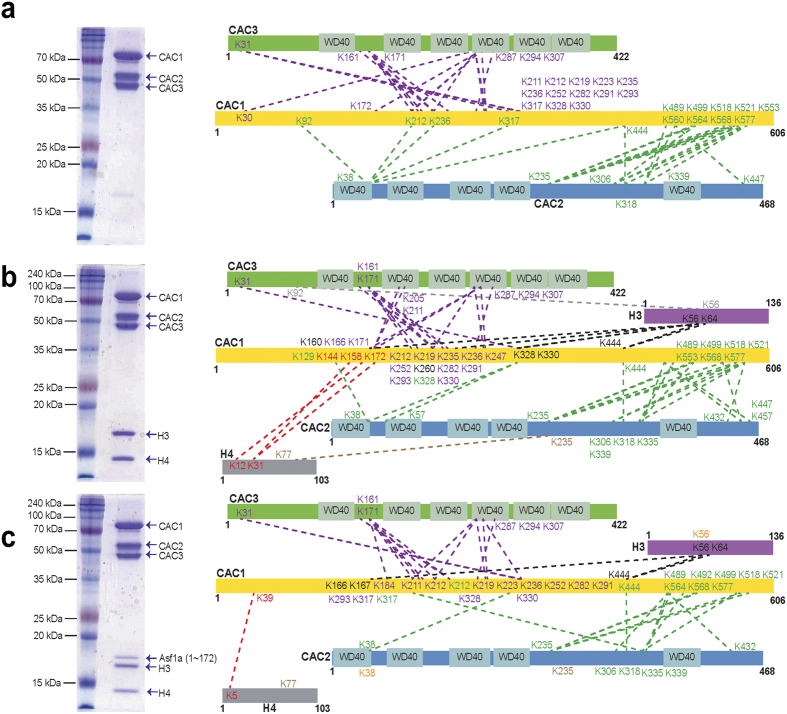
Cross-linking/mass spectrometry analysis on yCAF-1 complexes. (**a**) Purified yCAF-1 composed of Cac1, Cac2 and Cac3 is shown in a gel (left panel). Identified cross-link contacts are shown as dotted lines. (For clarity, only cross-links between different complex subunits are shown in different colors). (**b**) Same as A for the purified yCAF-1_H3/H4 complex. (**c**) Same as A for the yCAF-1_H3/H4_Asf1 complex.

To characterize the structural properties of the fully assembled CAF-1, we developed a baculovirus-expression based procedure to reconstitute the full three-subunit yCAF-1 (Fig. 1a). Using this approach, we were able to reconstitute and isolate a stable yCAF-1 complex composed of Cac1, Cac2 and Cac3. We first applied XL-MS using the amine-reactive reagent, disuccinimidyl suberate (DSS), to analyze the interaction network among yCAF-1 subunits (Fig. 1a and Supplementary Table 1)^14^. Our data revealed that both Cac2 and Cac3 are extensively cross-linked to Cac1, but no cross-links were found between Cac2 and Cac3. Cross-links between Cac1 and Cac3 were found in the middle region of Cac1 near the K/E/R rich domain (residues 135 to 225), while cross-links between Cac1 and Cac2 are found exclusively at the C-terminal region of Cac1.

**Figure 2:**
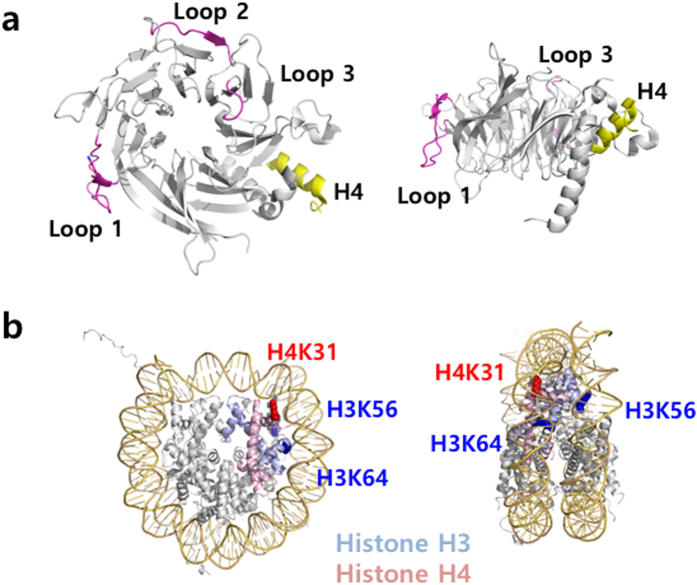
Structural analysis of the interactions between Cac3 and histones. (**a**) The cross-linked region of Cac3 with Cac1 was mapped on the structure of RbAp48 (3CFV), the human homologue of Cac3 (left panel in top view and right panel in side view). (**b**) The cross-linked residues between yCAF-1 and histones were mapped on the nucleosome structure (1AOI).

Two regions in Cac3 show the major crosslinks with Cac1 (Fig. 1a). The first region, which includes Lys161 and Lys171, is located within the loop between the 3^rd^ and 4^th^ β-strands of the second WD40 β-barrel blade (loop 1), based on sequence alignment and the previously published crystal structure of the homologous RbAp48. The second region, which includes Lys287, Lys294, and Lys307, corresponds to the loops located within the fourth and fifth blades of the β-barrel (loops 2 & 3) (Fig. 2). As these loops protrude away from the WD40 β-propeller structure of Cac3, we believe that some, if not all, of these residues would be involved in mediating interaction with Cac1. Our XL-MS data also showed that Cac2 is in close proximity to the C-terminal region of Cac1 and suggests that Cac2, similar to Cac3, likely interacts with Cac1 via loops between the blades of its predicted WD40 domain structure. Collectively, these results suggest that Cac1 uses two discrete regions to interact with Cac2 and Cac3, and to bridge Cac2 and Cac3 within the fully assembled CAF-1. Consistent with our XL-MS analysis on the interactions among Cac1, Cac2 and Cac3, previous *in-vitro* pulldown experiments with CAF-1 in other species have shown that that the C-terminal region of the largest subunit, human p150 or fly p180 interacts with the p60 subunit corresponding to Cac2^3^. To further verify that the middle region of Cac1 identified from our XL-MS analysis, interacts with Cac3, we utilized human CAF-1 complex and showed that the middle region of p150, (the largest subunit corresponding to Cac1) interacts with p48 (RbAP48, the smallest subunit of CAF-1 corresponding to Cac3) (Supplementary Fig. 1). The interactions analyzed by XL-MS among CAF-1 subunits together with *in-vitro* pulldown data show that that the largest subunit bridges the other two subunits in CAF-1.

We next analyzed the interactions among yCAF-1, histone H3/H4 and Asf1. We generated yCAF-1 complexes with histone H3/H4 (yCAF-1_H3/H4), or histone H3/H4 and Asf1 (yCAF-1_H3/H4_Asf1) and applied XL-MS using DSS to analyze the interaction network (Fig. 1a,b and Supplementary Tables 2 and 3). Compared with yCAF-1 complex alone, yCAF-1_H3/H4 and yCAF-1_H3/H4_Asf1 show similar patterns of cross-linking among yCAF-1 subunits, indicating that no major structural rearrangement among yCAF-1 subunits occurs upon binding of histones and Asf1. Our XL-MS analysis on yCAF-1_H3/H4 shows that the crosslinks between yCAF-1 and histone H3/H4 are found exclusively in Cac1. Specifically, the region encompassing residues 135-225, which includes the K/E/R rich domain, is mostly involved in interacting with histone H3 as well as H4 (Fig. 1b). On the other hand, XL-MS reveals minimal interactions between histones and other two subunits: Cac2 and Cac3. Previous studies have shown that human p150 interacts with histone Histone H3/H4 and that human p48 and fly p55 interact with the first helix of histone H4 as well as histone H3^6^,^7^,^15^. It is unclear why we did not identify crosslinks between histones, and Cac2 or Cac3. This might be due to limited number of solvent accessible lysines in Cac2 and Cac3 for crosslinking, which would affect XL-MS results. The smallest subunit of CAF-1 is a common component in several chromatin modifying complexes involved in distinct pathways including Polycomb Repressive Complex 2 (PRC2), Nucleosome Remodeling Deacetylase (NuRD), Nucleosome Remodeling Factor (NURF) and the HAT1 complex^16^,^17^,^18^,^19^,^20^,^21^,^22^. The protein binding pockets in human RbAp48 and fly p55 have been shown to be utilized differently depending on which complexes that this smallest subunit of CAF-1 resides in. Furthermore, histone tails is not necessary for CAF-1 to assemble nucleosome^23^. Therefore, alternatively, the histone binding mode in yCAF-1 might differ from what have been observed in previous studies conducted on individual protein subunits. These results suggest that the largest subunit, Cac1 may serve as a major interacting platform for histones H3 and H4, and other subunits might bind to histones transiently during nucleosome assembly. Cross-links in histone H3/H4 with yCAF-1 are limited to a few residues. Specifically, Lys56 and Lys64 of histone H3 were cross-linked to Lys444 in Cac1, and Lys12 and Lys31 of histone H4 were cross-linked to Cac1. Interestingly, the acetylation of histone H3 Lys56 and H4 Lys12 is found in newly-synthesized histone^24^,^25^,^26^. Moreover, the histone H3 Lys56 acetylation is shown to increase its binding affinity toward CAF-1^27^,^28^.

Our observation that Cac1 is in close proximity to histone H3 Lys56 and histone H4 Lys12 suggests that Cac1 might recognize the acetylated lysines in histone H3 and H4. Interestingly, in the context of the nucleosome structure, the histone lysine residues (K56 and K64 of histone H3, and K21 and K31 of histone H4) cross-linked to Cac1 are involved in interacting with DNA (Fig. 2b). In this binding mode, Cac1 would mask the DNA binding surface of the histone H3/H4 dimer and prevent nonspecific DNA binding toward highly charged histones during nucleosome assembly. Asf1 binds to H3/H4 via the hydrophobic interface between two histone H3/4 dimers, while CAF-1 interacts with the DNA binding surface of the histone H3/H4 tetramer^11^,^12^. This observation is consistent with the idea that Asf1 hands over the histone H3/H4 dimer to CAF-1. We did not detect any cross-linking between Asf1 and CAF-1 subunits in the XL-MS analysis of yCAF-1_histone H3/H4_Asf1. This might be due to the fact that we used the Asf1 core (residues 1 to 156) missing the C-terminal region, which are known to interact with Cac2^10^. Alternatively, the lack of crosslink could be attributed to the scarcity of lysines within the cross-linking distance near the hydrophobic interface between the Asf1 core and histone H3/H4. Interestingly, we observed that the number of the cross-links between histones and Cac1 is substantially lower in yCAF-1_H3/H4_Asf1 complex than in yCAF-1_H3/H4 complex. This observation raises an interesting possibility that Asf1 binding induces conformational change in histone H3/H4 to reduce the accessibility of the Cac1 binding interface for crosslinking. This is also consistent with a previous study showing that p48 binding to histone H3/H4 induces conformational change in histone H3/H4^13^. Alternatively, the binding of Asf1 to histone H3/H4 may partially dissociate CAF-1 from histone H3/H4, as a previous study showed that the binding of p48 to H3/H4 weakens the interaction between Asf1 and histone H3/H4^13^. However, as we have observed stable complex formation of CAF-1_H3/H4_Asf1 (Fig. 1c), at this stage, we cannot distinguish these two possibilities. It is possible that we might have captured a transition state during the handover of histone H3/H4 from Asf1 to CAF-1.

### Single-particle EM analysis on yCAF-1 as a whole complex with histones and Asf1 chaperon

**Figure 3:**
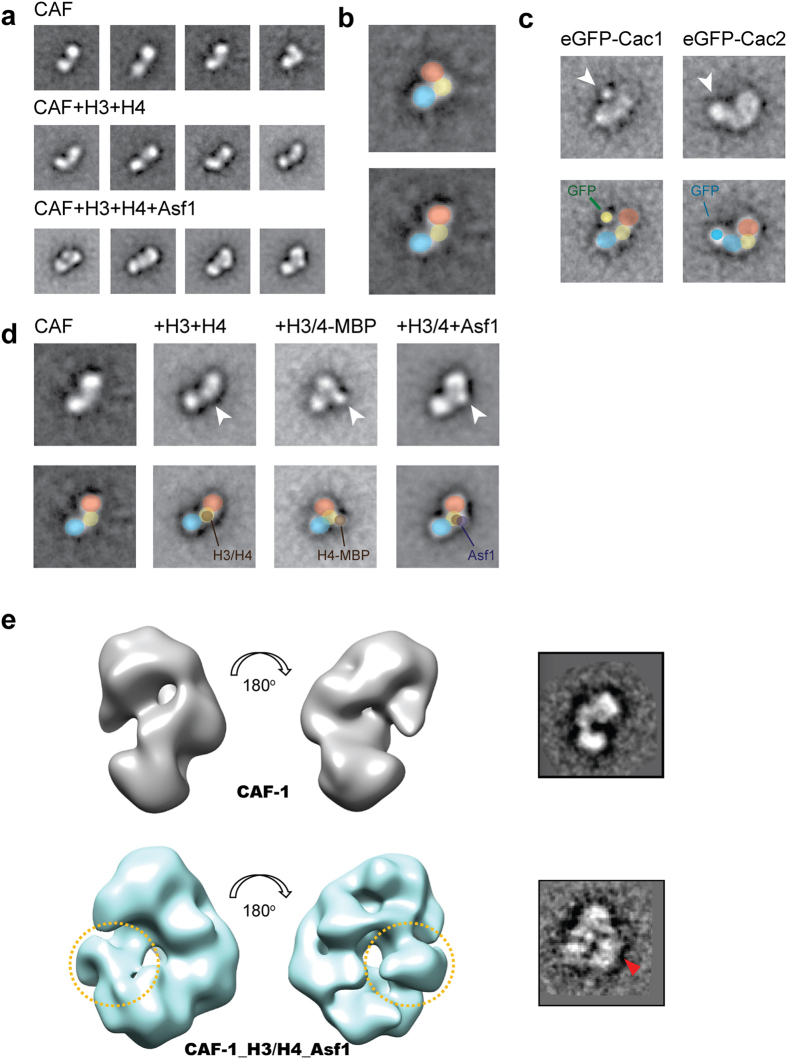
Single-particle electron microscopy (EM) analysis of the yCAF-1 complex. (**a**) Representative class averages of negatively stained, GraFix stabilized yCAF-1, yCAF-1_H3/H4, and yCAF-1_H3/H4_Asf1 complexes. (**b**) Proposed organization of the 2D class average into three distinct regions marked with different colors. (**c**) Localization of the relative positions of Cac1 and Cac2. yCAF-1 with Cac1 or Cac2 fused to N-terminal eGFP tags were visualized. Additional density compared to the untagged complex are marked by white arrowheads. (**d**) Localization of H3/H4 and Asf1 binding. Additional density in the complexes containing H3/H4 and Asf1 compared to yCAF-1 alone are denoted by white arrowheads. +H3/4-MBP corresponds to yCAF-1 complex with histone H3 and the N-terminal 20 residues of histone H4 fused to maltose binding protein. (**e**) 3D reconstructed EM structures of CAF-1 alone and CAF-1_H3/H4_Asf1. Two different views with 180° rotation are shown in right panel for CAF-1 alone (upper left) and CAF-1_H3/H4_Asf1 (lower left). The extra-density corresponding to H3/H4_Asf1 is indicated by yellow dotted circles. 2D class averages corresponding to the view of 3D reconstructions are shown in the right panels and the position of H3/H4_Asf1 is indicated by a red triangle.

To gain insights into the structure of yCAF-1, we examined the reconstituted yCAF-1 by negative stain single-particle EM with the random conical tilt approach (Fig. 3a)^29^. The two-dimensional (2D) class averages revealed the purified complex has extensive structural heterogeneity (Supplementary Fig. 2) indicating that there is conformational flexibility among the subunits. In order to mitigate this heterogeneity, we stabilized yCAF-1 using the gradient fixation technique^30^. The 2D class averages of yCAF-1 shows an overall bilobal shape with two lobes connected by a middle ‘hinge’ region (Fig. 3a,b). To further dissect the subunit organization of yCAF-1, we applied an established EM-based labeling approach. We first generated yCAF-1 where eGFP is fused to the N-terminus of Cac1 (eGFP-Cac1) and obtained class averages (Fig. 3c). The class averages of yCAF-1 containing eGFP-Cac1 show extra density located between the two lobes. We next generated yCAF-1 where eGFP is fused at the N-terminus of Cac2 (eGFP-Cac2). The class averages of yCAF-1 containing eGFP-Cac2 clearly show extra density at one of two lobes indicating that one of the lobes corresponds to Cac2 (Fig. 3c). Unfortunately, we were not able to observe any extra density from yCAF-1 containing eGFP-Cac3, precluding the unambiguous localization of Cac3. This might due to the flexibility of the eGFP linker fused at the N-terminal of Cac3. The two lobe regions are relatively invariable between class averages, and are consistent in size and shape. Considering that both Cac2 and Cac3 adopt similar WD40-repeat β-propeller structures, it is most likely that the other lobe in the EM structure corresponds to Cac3. Conversely, the middle hinge region varies between different class averages (Fig. 3a), consistent with a flexible and disordered structure that Cac1 is predicted to have. These observations suggest that two lobe densities correspond to Cac2 and Cac3 connected by Cac1. This interpretation is also supported by our XL-MS analysis that Cac2 and Cac3 interact with two distinct regions of Cac1 but not with one another.

We next analyzed yCAF-1 H3/H4, and yCAF-1_H3/H4_Asf1 (Fig. 3d). The 2D class averages show extra density between two lobes compared with the class averages of CAF-1 alone, suggesting that the interface between two lobes might be the histone binding groove. Since the human (p48) and fly (p55/Nurf55) homologues of Cac3 have been shown to interact with the first helix of histone H4^6^,^7^, we tried to map the histone binding site by taking advantage of this known interaction. More specifically, we reconstituted yCAF-1 in complex with maltose binding protein fused H4 N-terminal peptide (residues 1 to 40, the first helix of histone H4) and analyzed this complex by negative stain EM (Fig. 3d). The class averages of yCAF-1_MBP-H4 show extra density between two lobes supporting the hypothesis that the groove formed by Cac2 and Cac3 accommodates histone H3/H4.

To further investigate the structure of yCAF-1, we collected a separate dataset from negatively stained samples prepared by GraFix and determined the 3D reconstructions of yCAF-1 alone and yCAF-1_H3/H4_Asf1 complex at around 30 Å resolution (Fig. 3e,f, and Supplementary Fig. 3). The overall structural features of these two 3D reconstructions are fully consistent with the 2D class averages that we generated earlier from a separate dataset, validating these newly determined structures. When we compared the 3D structures of yCAF-1 alone and yCAF-1_H3/H4_Asf1, we could clearly observe extra density corresponding the histone H3/H4_Asf1 between the two lobes that we proposed to be Cac2 and Cac3, confirming our earlier hypothesis that Cac1 generates the critical binding platform for histones.

In summary, our studies provided the first structural insights into the full yCAF-1 alone as well as in complex with histone H3/H4 and Asf1. Structural analysis on yCAF-1 reveals that it adopts a bilobal shape with a flexible hinge, and further EM analysis in combination with XL-MS data shows that Cac1 may form a histone binding groove between the two lobes representing Cac2 and Cac3, respectively. Interestingly, Cac1 interacts with the DNA binding surface of histone H3/H4 dimer (Fig. 2b). Considering that Asf1 interacts with the hydrophobic tetramer interface of histone H3/H4, which is located at the opposite side of the DNA binding surface (Fig. 2b), this configuration would allow Asf1 to hand over histone H3/H4 to the CAF-1 complex by maintaining a minimal interaction between Asf1 and CAF-1. Overall, this work presents the first structural glimpse into the molecular architecture of CAF-1 complex and generates a structural framework to further dissect the complex mechanism of chromatin assembly.

## Methods

### yCAF-1 reconstitution and purification

Sf9 cells were cultured in HyClone^TM^ CCM3^TM^ Media (GE Healthcare Life Sciences). CAF-1 genes were delivered to Sf9 cells using P3 baculovirus, generated using Baculovirus Expression Vector System prior to infection. Cell density was around 2.0 ~ 2.5 × 10^6^ cells/ml during infection and infected cells were incubated at 27 °C for 48 ~ 72 hours. Cells were harvested using lysis buffer (100 mM NaCl, 50 mM Tris-HCl pH 8.0 and 5% glycerol). CAF-1 was purified from cell extract first by Ni-NTA (Nickel-Nitrilotriacetic acid agarose, Qiagen) resin. The complex was eluted using 100 mM imidazole-containing lysis buffer. Purity of the complex was improved by ion exchange chromatography using HiTrap^TM^ Q HP (GE Healthcare Life Sciences) and size exclusion chromatography using HiLoad 26/60 Superdex 200 prep grade in sequence. Finally, the complex was concentrated using Amicon Ultra-15 Centrifugal Filter Units (Millipore) and analyzed by SDS-PAGE and Coomassie blue staining.

### Reconstitution of the complexes

Several different CAF-1 related complexes were reconstituted for EM and XL-MS analysis. For CAF-1_H3/H4_Asf1a complex, Asf1a with amino acids from 1 to 172 was first reconstituted with Histone H3-H4 (*Xenopus laevis*) dimer in dialysis buffer (20 mM Tris-HCl pH 7.5, 500 mM NaCl, 5 mM 2-mercaptoethanol). Dialyzed Asf1a-H3-H4 was subjected to size exclusion chromatography using HiLoad 26/60 Superdex 200 prep grade to obtain a stoichiometrically and structurally homogenous complex. Asf1a-H3-H4 and CAF-1 were reconstituted (50 mM Tris-HCl pH 8.0, 150 mM NaCl, 10% glycerol, 20 mM 2-mecaptoethanol) using HiLoad 26/60 Superdex 200 prep grade. Likewise, CAF-1_H3-H4, CAF-1_MBP-H4 and GFP fused CAF-1 complexes were prepared. Asf1a and MBP-H4 were expressed in BL21-CodonPlus (DE3)-RIPL competent cells prior to reconstitution.

### Cross-linking/mass spectrometry

XL-MS experiments on the complexes, including cross-linking with DSS-d_0_/d_12_ (Creative Molecules), MS analysis on a LTQ Orbitrap XL mass spectrometer (Thermo) and data processing with xQuest were performed as described^14^.

### Gradient fixation of CAF-1

Prior to EM imaging, purified CAF-1 complex was subjected to gradient fixation as previously described^31^. Briefly, a continuous 12–24% glycerol and 0–0.05% glutaraldehyde gradient (150 mM NaCl, 50 mM HEPES pH 7.4) was generated using a Gradient Master (Biocomp). Purified complexes were applied to the top of the gradient and centrifuged at 40,000 rpm for 20 hours (Beckman SW-55 rotor). The gradients were fractionated using a Gradient Master (Biocomp). Fractions containing CAF-1 was identified by following an identical gradient lacking glutaraldehyde centrifuged in parallel from silver-stained SDS-PAGE gels.

### Electron microscopy

Negative-stained EM samples were generated as described^32^. Samples were imaged using a Tecnai Spirit transmission electron microscope (FEI) operated at an accelerating voltage of 120 kV. Images were obtained at a nominal magnification of 49,000× using an FEI Eagle 4 K × 4 K charge-coupled device camera at a defocus of −1.2 μm. Images of tilt pairs were taken at 0° and 60°. 2 × 2 image pixels were averaged for a final pixel size of 4.67 Å. Individual particle images were selected manually using Boxer^33^. Single particle images were then aligned, sorted using K-means classification, and averaged using the SPIDER imaging suite. Representative class averages are presented, and full galleries of the complexes without N-terminal tags are shown in Supplementary Fig. 1. Datasets for yCAF-1, yCAF-1_H3/H4, and yCAF-1_H3/H4_Asf1 contain 4260, 5044, and 8735 particles, respectively. For 3D reconstruction, purified CAF-1 complex was applied to ultracentrifugation at 74,329× g for 16 hours with a 5–20% sucrose gradient in presence of a 0–0.2% glutaraldehyde gradient. A fraction containing only the CAF-1 complex was collected and then negatively stained with 2% (w/v) uranyl acetate for 1 min on 400 mesh carbon grids. Images were collected at 60,000× magnification with a defocus value of 0.8–2.0 μm on a 4 × 4 K CCD camera (Tietz Vieo and imaging Processing System) attached to a Jeol JEM2100F filed emission gun transmission electron microscope at 200 kV (Supplementary Fig. 5). Data were further processed using EMAN2 program^33^. To reconstitute CAF-1/Asf1/H3/H4 complex, 2-fold excessive amount of Asf1/H3/H4 subcomplex were mixed with CAF-1 in binding buffer containing 20 mM Tris pH 7.5, 200 mM NaCl, and 2 mM β-mercaptoethanol. The mixture incubated at 4 °C for 1 hour and then applied to ultracentrifugation as described above. The complex fractions were collected and concentrated using Vivaspin 500 (GE healthcare). In total, 10,306 and 4,990 particles were picked for yCAF-1 and the yCAF-1/Asf1/H3/H4 complex, respectively. The selected particles were used for further processing to generate reference-free 2D class-averages. 44 and 31 representative 2D classes were selected from each 2D class stack and used to build initial models. Selected models were further iteratively refined with low-pass-filter (cutoff = 0.033) using EMAN2. Entire yCAF-1_H3H4_Asf1 processing was totally independent from yCAF-1 model.

## Additional Information

**How to cite this article**: Kim, D. *et al.* Molecular Architecture of Yeast Chromatin Assembly Factor 1. *Sci. Rep.* **6**, 26702; doi: 10.1038/srep26702 (2016).

## Supplementary Material

Supplementary Information

## References

[b1] Ransom M., Dennehey B. K. & Tyler J. K. Chaperoning histones during DNA replication and repair. Cell 140, 183–95 (2010). 20141833 10.1016/j.cell.2010.01.004 PMC3433953

[b2] Ramirez-Parra E. & Gutierrez C. The many faces of chromatin assembly factor 1. Trends Plant Sci 12, 570–6 (2007). 17997123 10.1016/j.tplants.2007.10.002

[b3] Kaufman P. D., Kobayashi R., Kessler N. & Stillman B. The p150 and p60 subunits of chromatin assembly factor I: a molecular link between newly synthesized histones and DNA replication. Cell 81, 1105–14 (1995). 7600578 10.1016/s0092-8674(05)80015-7

[b4] Tyler J. K. *et al.* Interaction between the Drosophila CAF-1 and ASF1 chromatin assembly factors. Mol Cell Biol 21, 6574–84 (2001). 11533245 10.1128/MCB.21.19.6574-6584.2001 PMC99803

[b5] Takami Y., Ono T., Fukagawa T., Shibahara K. & Nakayama T. Essential role of chromatin assembly factor-1-mediated rapid nucleosome assembly for DNA replication and cell division in vertebrate cells. Mol Biol Cell 18, 129–41 (2007). 17065558 10.1091/mbc.E06-05-0426 PMC1751324

[b6] Song J. J., Garlick J. D. & Kingston R. E. Structural basis of histone H4 recognition by p55. Genes Dev 22, 1313–8 (2008). 18443147 10.1101/gad.1653308 PMC2377184

[b7] Murzina N. V. *et al.* Structural basis for the recognition of histone H4 by the histone-chaperone RbAp46. Structure 16, 1077–85 (2008). 18571423 10.1016/j.str.2008.05.006 PMC2572730

[b8] Moggs J. G. *et al.* A CAF-1-PCNA-mediated chromatin assembly pathway triggered by sensing DNA damage. Mol Cell Biol 20, 1206–18 (2000). 10648606 10.1128/mcb.20.4.1206-1218.2000 PMC85246

[b9] Mello J. A. *et al.* Human Asf1 and CAF-1 interact and synergize in a repair-coupled nucleosome assembly pathway. EMBO Rep 3, 329–34 (2002). 11897662 10.1093/embo-reports/kvf068 PMC1084056

[b10] Malay A. D., Umehara T., Matsubara-Malay K., Padmanabhan B. & Yokoyama S. Crystal structures of fission yeast histone chaperone Asf1 complexed with the Hip1 B-domain or the Cac2 C terminus. J Biol Chem 283, 14022–31 (2008). 18334479 10.1074/jbc.M800594200

[b11] English C. M., Adkins M. W., Carson J. J., Churchill M. E. & Tyler J. K. Structural basis for the histone chaperone activity of Asf1. Cell 127, 495–508 (2006). 17081973 10.1016/j.cell.2006.08.047 PMC2981792

[b12] Natsume R. *et al.* Structure and function of the histone chaperone CIA/ASF1 complexed with histones H3 and H4. Nature 446, 338–41 (2007). 17293877 10.1038/nature05613

[b13] Zhang W. *et al.* Structural plasticity of histones H3-H4 facilitates their allosteric exchange between RbAp48 and ASF1. Nat Struct Mol Biol 20, 29–35 (2013). 23178455 10.1038/nsmb.2446 PMC3538076

[b14] Leitner A., Walzthoeni T. & Aebersold R. Lysine-specific chemical cross-linking of protein complexes and identification of cross-linking sites using LC-MS/MS and the xQuest/xProphet software pipeline. Nat Protoc 9, 120–37 (2014). 24356771 10.1038/nprot.2013.168

[b15] Schmitges F. W. *et al.* Histone methylation by PRC2 is inhibited by active chromatin marks. Mol Cell 42, 330–41 (2011). 21549310 10.1016/j.molcel.2011.03.025

[b16] Smith S. & Stillman B. Purification and characterization of CAF-I, a human cell factor required for chromatin assembly during DNA replication *in vitro*. Cell 58, 15–25 (1989). 2546672 10.1016/0092-8674(89)90398-x

[b17] Tyler J. K., Bulger M., Kamakaka R. T., Kobayashi R. & Kadonaga J. T. The p55 subunit of Drosophila chromatin assembly factor 1 is homologous to a histone deacetylase-associated protein. Mol Cell Biol 16, 6149–59 (1996). 8887645 10.1128/mcb.16.11.6149 PMC231618

[b18] Verreault A., Kaufman P. D., Kobayashi R. & Stillman B. Nucleosome assembly by a complex of CAF-1 and acetylated histones H3/H4. Cell 87, 95–104 (1996). 8858152 10.1016/s0092-8674(00)81326-4

[b19] Martinez-Balbas M. A., Tsukiyama T., Gdula D. & Wu C. Drosophila NURF-55, a WD repeat protein involved in histone metabolism. Proc Natl Acad Sci USA 95, 132–7 (1998). 9419341 10.1073/pnas.95.1.132 PMC18150

[b20] Wade P. A., Jones P. L., Vermaak D. & Wolffe A. P. A multiple subunit Mi-2 histone deacetylase from Xenopus laevis cofractionates with an associated Snf2 superfamily ATPase. Curr Biol 8, 843–6 (1998). 9663395 10.1016/s0960-9822(98)70328-8

[b21] Czermin B. *et al.* Drosophila enhancer of Zeste/ESC complexes have a histone H3 methyltransferase activity that marks chromosomal Polycomb sites. Cell 111, 185–96 (2002). 12408863 10.1016/s0092-8674(02)00975-3

[b22] Muller J. *et al.* Histone methyltransferase activity of a Drosophila Polycomb group repressor complex. Cell 111, 197–208 (2002). 12408864 10.1016/s0092-8674(02)00976-5

[b23] Shibahara K., Verreault A. & Stillman B. The N-terminal domains of histones H3 and H4 are not necessary for chromatin assembly factor-1-mediated nucleosome assembly onto replicated DNA *in vitro*. Proc Natl Acad Sci USA 97, 7766–71 (2000). 10884407 10.1073/pnas.97.14.7766 PMC16619

[b24] Allis C. D., Chicoine L. G., Richman R. & Schulman I. G. Deposition-related histone acetylation in micronuclei of conjugating Tetrahymena. Proc Natl Acad Sci USA 82, 8048–52 (1985). 3865215 10.1073/pnas.82.23.8048 PMC391439

[b25] Sobel R. E., Cook R. G. & Allis C. D. Non-random acetylation of histone H4 by a cytoplasmic histone acetyltransferase as determined by novel methodology. J Biol Chem 269, 18576–82 (1994). 8034606

[b26] Sobel R. E., Cook R. G., Perry C. A., Annunziato A. T. & Allis C. D. Conservation of deposition-related acetylation sites in newly synthesized histones H3 and H4. Proc Natl Acad Sci USA 92, 1237–41 (1995). 7862667 10.1073/pnas.92.4.1237 PMC42674

[b27] Winkler D. D., Zhou H., Dar M. A., Zhang Z. & Luger K. Yeast CAF-1 assembles histone (H3-H4)2 tetramers prior to DNA deposition. Nucleic Acids Res 40, 10139–49 (2012). 22941638 10.1093/nar/gks812 PMC3488248

[b28] Li Q. *et al.* Acetylation of histone H3 lysine 56 regulates replication-coupled nucleosome assembly. Cell 134, 244–55 (2008). 18662540 10.1016/j.cell.2008.06.018 PMC2597342

[b29] Radermacher M., Wagenknecht T., Verschoor A. & Frank J. Three-dimensional reconstruction from a single-exposure, random conical tilt series applied to the 50S ribosomal subunit of Escherichia coli. J Microsc 146, 113–36 (1987). 3302267 10.1111/j.1365-2818.1987.tb01333.x

[b30] Kastner B. *et al.* GraFix: sample preparation for single-particle electron cryomicroscopy. Nat Methods 5, 53–5 (2008). 18157137 10.1038/nmeth1139

[b31] Stark H. GraFix: stabilization of fragile macromolecular complexes for single particle cryo-EM. Methods Enzymol 481, 109–26 (2010). 20887855 10.1016/S0076-6879(10)81005-5

[b32] Ohi M., Li Y., Cheng Y. & Walz T. Negative Staining and Image Classification - Powerful Tools in Modern Electron Microscopy. Biol Proced Online 6, 23–34 (2004). 15103397 10.1251/bpo70 PMC389902

[b33] Ludtke S. J., Baldwin P. R. & Chiu W. EMAN: semiautomated software for high-resolution single-particle reconstructions. J Struct Biol 128, 82–97 (1999). 10600563 10.1006/jsbi.1999.4174

