# Supplementary material for: Molecular Architecture of Yeast Chromatin Assembly Factor 1
Source: Sci Rep. 2016 May 25;6:26702. doi: 10.1038/srep26702 (PMC4879628; doi:10.1038/srep26702)
Supplement: Supplementary Information [file srep26702-s1.pdf]

Molecular Architecture of Yeast Chromatin Assembly Factor 1

Daegeun Kim[1,*], Dheva Setiaputra[2,*], Taeyang Jung[1,3,*], Jaehee Chung[1], Alexander Leitner[4], Jungmin Yoon[1], Ruedi Aebersold[4,5], Hans Hebert[3], Calvin K. Yip[2,#], Ji-Joon Song[1,#]

[1]Department of Biological Sciences, KAIST Institute for the BioCentury, Cancer Metastasis Control Center, KAIST, 291 Daehakro Yuseong Daejeon, 34141, Korea.
[2]Department of Biochemistry and Molecular Biology, The University of British Columbia, Vancouver, British Columbia, Canada, V6T 1Z3.
[3]Department of Biosciences and Nutrition, Karolinska Institute, and School of Technology and Health, KTH Royal Institute of Technology, Novum, SE-141 57, Sweden
[4]Department of Biology, Institute of Molecular Systems Biology, ETH Zurich, 8093 Zurich , Switzerland.
[5]Faculty of Science, University of Zurich, Zurich, Switzerland.
* These authors contributed equally.
[#] Correspondence: calvin.yip@ubc.ca & songj@kaist.ac.kr

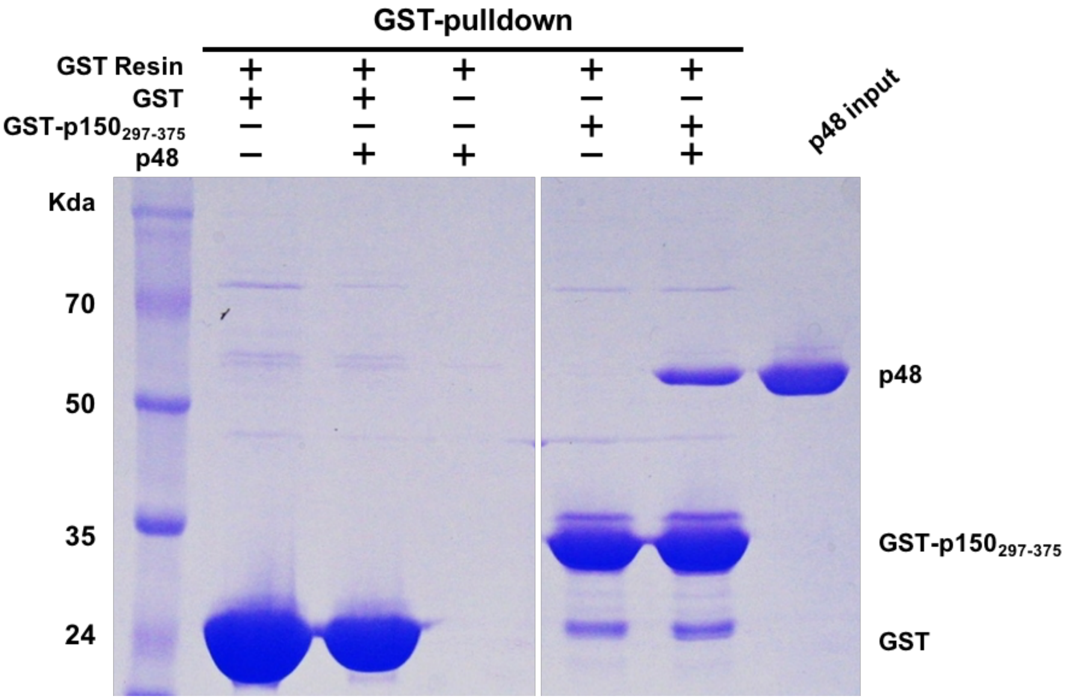

Supplementary Figure 1. GST-pull down experiments showing that the middle region of the largest subunit interacts with p48 of Caf-1.

The middle region of the largest subunit of human CAF-1 ($p150_{297\text{-}375}$) was generated in GST fusion, and incubated with p48. p48 specifically interact with the middle region of p150 but not GST.

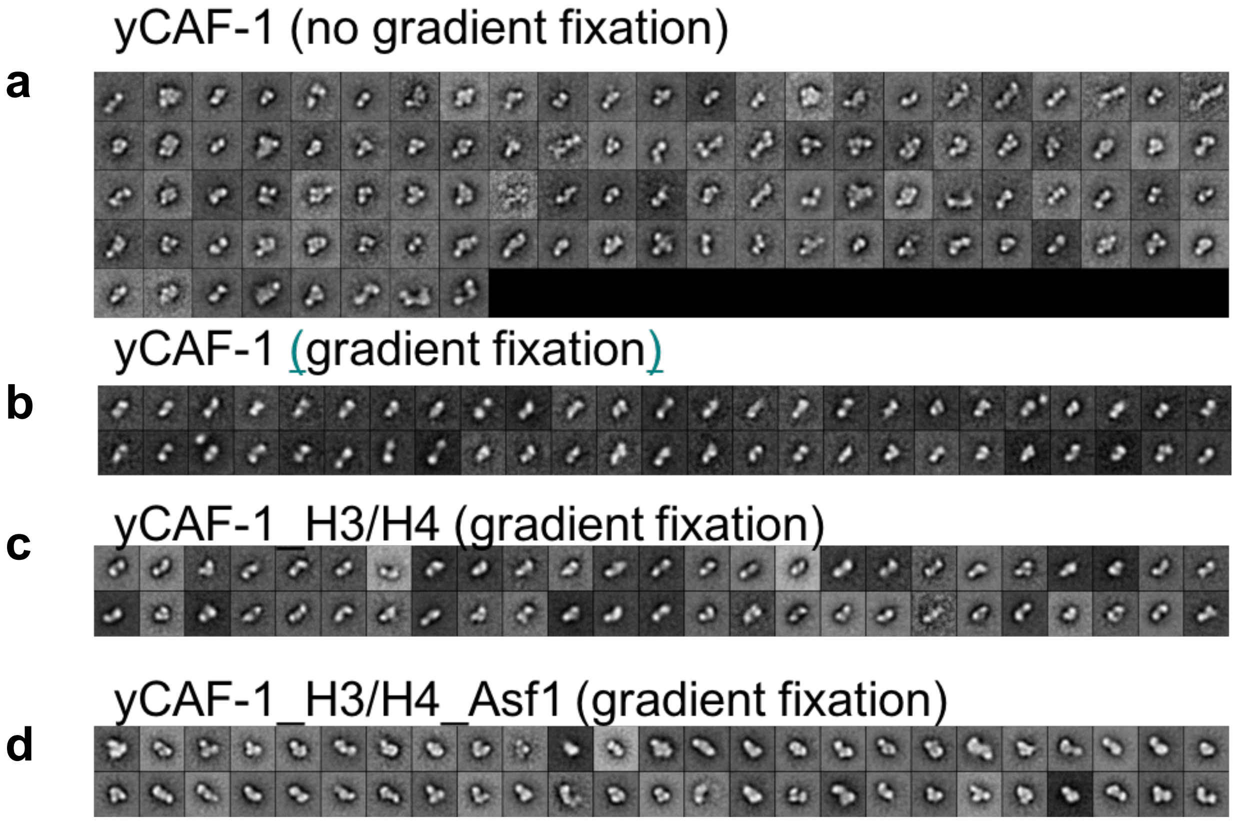

Supplementary Figure 2. Galleries of yCAF-1 class averages from 2D negative stain EM analysis.

(a) yCAF-1 complex prepared without gradient fixation stabilization (4,398 particles). Side length of all class averages shown is 374 Å. (b,c,d) yCAF-1 (4,260 particles), yCAF-1_H3/H4 (5,044 particles), and yCAF-1_H3/H4_Asf1 complexes (8,735 particles) prepared with gradient fixation stabilization. Side length for these class averages is 336 Å.

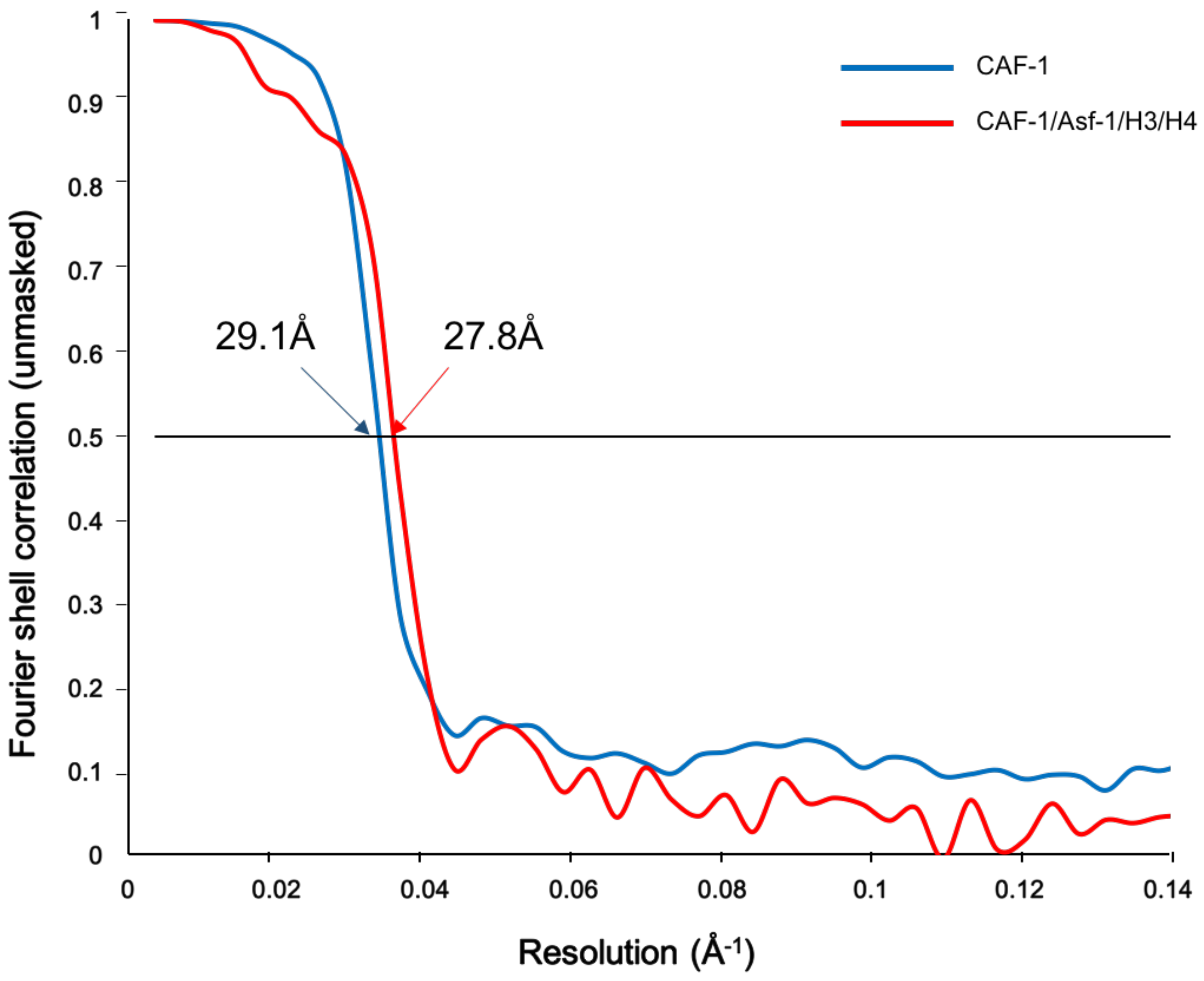

Supplementary Figure 3. Fourier Shell Correlation Curves.

FSC for CAF-1 (Blue) and CAF-1_H3/H4_Asf-1 (Red) indicates the resolution for the 3D reconstructions with 29.1Å and 27.8 Å respectively

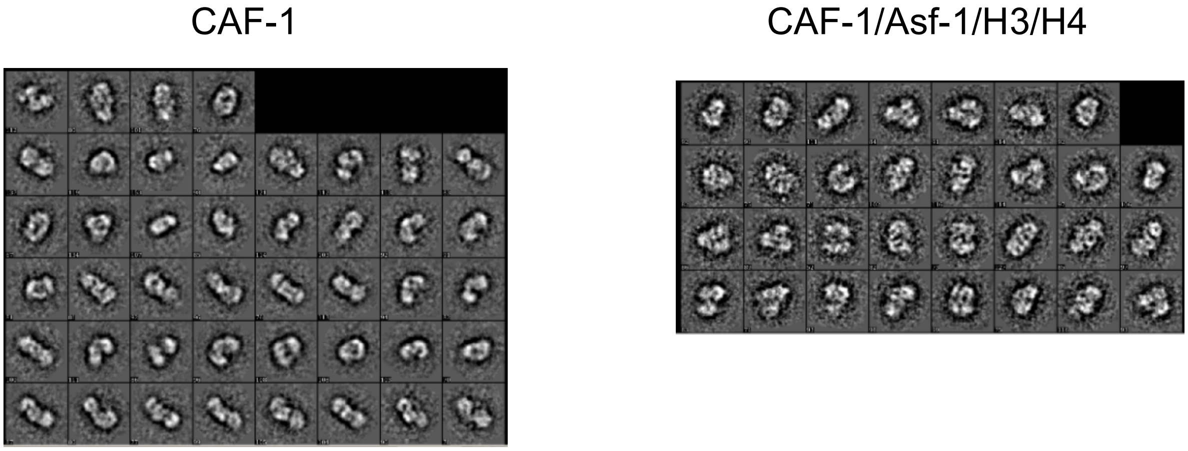

Supplementary Figure 4. 2D class averages for yCAF-1 and yCAF-1_H3/H4_Asf-1 used for 3D reconstruction.

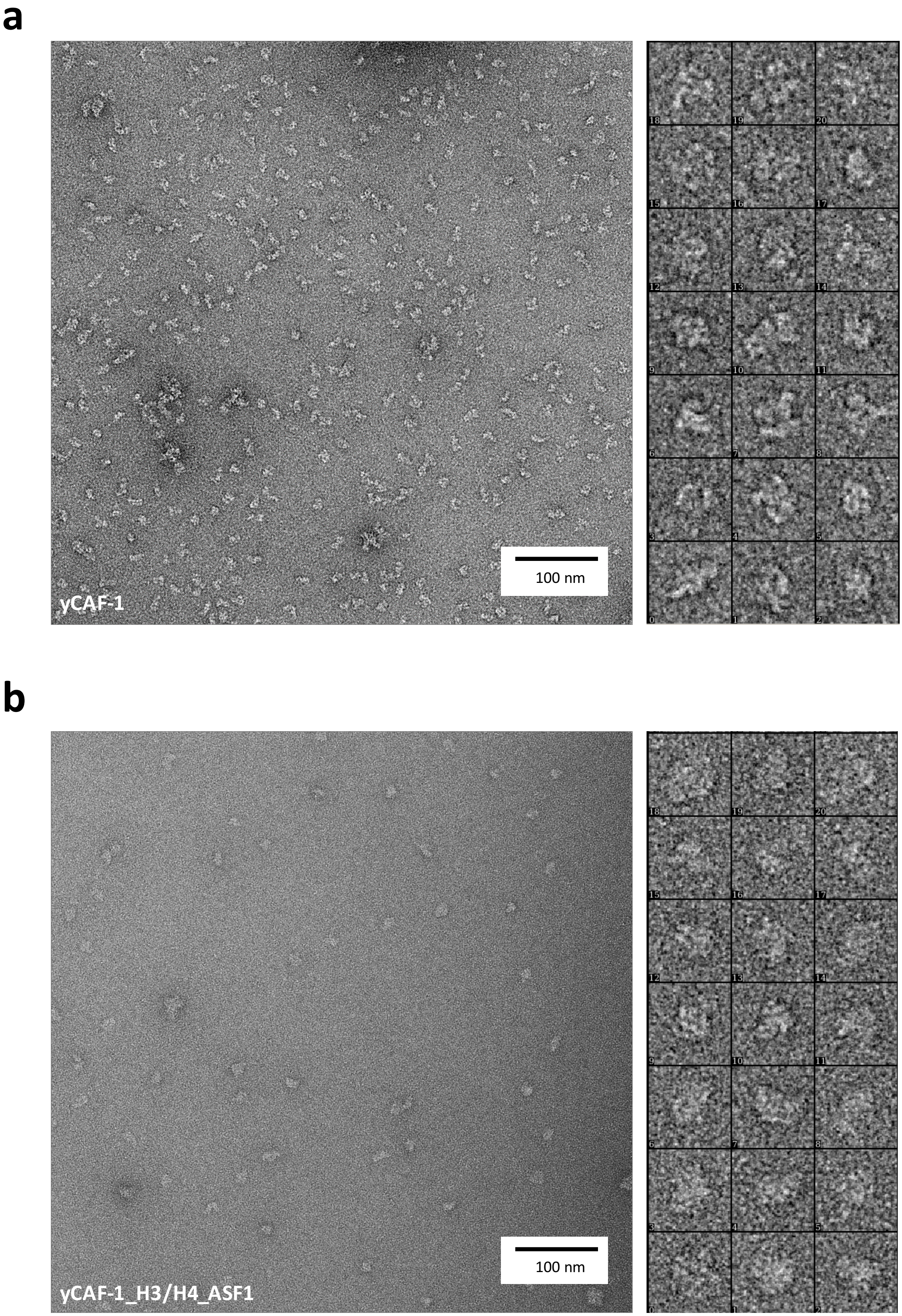

Supplementary Figure 5

Representative micrographs and particles of yCAF-1 (a) and yCAF-1_H3/H4_ASF1 complex (b)

Supplementary Table 1. Cross-linked peptides of yeast CAF-1

Cross-linked Peptide ID	Protein1	Protein2	Position1	Position2
GILSFFQNTTTVKSNK-KEKLQSNR-a13-b3	CAC1	CAC3	30	287
LLCYKNSPIQSTK-LFTAGGDNKVR-a5-b9	CAC1	CAC2	92	38
KLSDSNTPVVEK-LFTAGGDNKVR-a1-b9	CAC1	CAC2	236	38
KLSDSNTPVVEK-TKHGSTR-a1-b2	CAC1	CAC3	236	161
KLSDSNTPVVEK-EKLQSNR-a1-b2	CAC1	CAC3	236	287
KLSDSNTPVVEK-QSKISHPFETK-a1-b3	CAC1	CAC3	236	171
SDYEKFFLPFYAK-QSKISHPFETK-a5-b3	CAC1	CAC3	252	171
IDDELLNSKDK-EKLQSNR-a9-b2	CAC1	CAC3	291	287
KIDDELLNSKDK-EKLQSNR-a10-b2	CAC1	CAC3	291	287
DKTSSDDLLNWLQSR-EKHDGGVNSCR-a2-b2	CAC1	CAC3	293	294
DKTSSDDLLNWLQSR-EKLQSNR-a2-b2	CAC1	CAC3	293	287
KAVDVLQQMPLK-LFTAGGDNKVR-a1-b9	CAC1	CAC2	317	38
RKAVDVLQQMPLK-LFTAGGDNKVR-a2-b9	CAC1	CAC2	317	38
EKTDDELQSLLAQVPHK-NTKLLYDYLNTNSTK-a2-b3	CAC1	CAC3	330	31
KFVGPLIPTICLK-LFTAGGDNKVR-a1-b9	CAC1	CAC2	444	38
AEVIIETDGPIDPFKEPK-SVLKLPYK-a15-b4	CAC1	CAC2	489	339
SNSDLQAQTASQSQSPEKK-IAKAELPCPGDVLR-a18-b3	CAC1	CAC2	518	235
LFDGVQDSTFSLGTVTEIAQKNLPQYNK-IAKAELPCPGDVLR-a21-b3	CAC1	CAC2	553	235
SSGKGDLPR-SVLKLPYK-a4-b4	CAC1	CAC2	577	339
LFTAGGDNKVR-LKKEEEIR-a9-b3	CAC2	CAC1	38	212
IAKAELPCPGDVLR-SSGKGDLPR-a3-b4	CAC2	CAC1	235	577
IAKAELPCPGDVLR-NTIKEYAIR-a3-b4	CAC2	CAC1	235	568
IAKAELPCPGDVLR-QTIKNTIK-a3-b4	CAC2	CAC1	235	564
IAKAELPCPGDVLR-QKAMITDPMDLLR-a3-b2	CAC2	CAC1	235	521
IAKAELPCPGDVLR-NLPQYNKQTIK-a3-b7	CAC2	CAC1	235	560

SGILNSAGGVKNRPAIR-QKAMITDPMDLLR-a11-b2	CAC2	CAC1	306	521
SGILNSAGGVKNRPAIR-SSGKGDLPR-a11-b4	CAC2	CAC1	306	577
SGILNSAGGVKNRPAIR-NTIKEYAIR-a11-b4	CAC2	CAC1	306	568
SGILNSAGGVKNRPAIR-SSGKGDLPRK-a11-b4	CAC2	CAC1	306	577
KPALMAAFSPVFYETCQK-KFVGPLIPTICLK-a1-b1	CAC2	CAC1	318	444
KPALMAAFSPVFYETCQK-SSGKGDLPR-a1-b4	CAC2	CAC1	318	577
KPALMAAFSPVFYETCQK-RKFVGPLIPTICLK-a1-b2	CAC2	CAC1	318	444
SVLKLPYK-TSSLPSKR-a4-b7	CAC2	CAC1	339	499
EAGGIVNMLPVKK-TSSLPSKR-a12-b7	CAC2	CAC1	447	499
NTKLLYDYLNTNSTK-KAVDVLQQMPLK-a3-b1	CAC3	CAC1	31	317
NTKLLYDYLNTNSTK-AVDVLQQMPLKEK-a3-b11	CAC3	CAC1	31	328
QSKISHPFETK-LKEEAK-a3-b2	CAC3	CAC1	171	219
QSKISHPFETK-LKEEAKER-a3-b6	CAC3	CAC1	171	223
QSKISHPFETK-IGNFFKK-a3-b6	CAC3	CAC1	171	235
QSKISHPFETK-LKKEEEIR-a3-b3	CAC3	CAC1	171	212
EKHDGGVNSCR-LKKEEEIR-a2-b2	CAC3	CAC1	294	211
EKHDGGVNSCR-LKKEEEIR-a2-b3	CAC3	CAC1	294	212
EKHDGGVNSCR-ELKVEEER-a2-b3	CAC3	CAC1	294	172
FNYKNSLILASADSNGR-KIDDELLNSK-a4-b1	CAC3	CAC1	307	282
FNYKNSLILASADSNGR-RKIDDELLNSK-a4-b2	CAC3	CAC1	307	282

Supplementary Table 2. Cross-linked peptides of yeast CAF-1 _HistoneH3/H4

Cross-linked Peptide ID	Protein1	Protein2	Position1	Position2
KLSDSNTPVVEK-TKHGSTR-a1-b2	CAC1	CAC3	236	161
KLSDSNTPVVEK-EKLQSNR-a1-b2	CAC1	CAC3	236	287
KLSDSNTPVVEKSDYEK-QSKISHPFETK-a12-b3	CAC1	CAC3	247	171
SDYEKFFLPFYAK-QSKISHPFETK-a5-b3	CAC1	CAC3	252	171
FFLPFYAKDGVR-YQKSTELLIR-a8-b3	CAC1	H3	260	57
IDDELLNSKDK-EKLQSNR-a9-b2	CAC1	CAC3	291	287
KIDDELLNSKDK-EKLQSNR-a10-b2	CAC1	CAC3	291	287
DKTSSDDLLNWLQSR-EKHDGGVNSCR-a2-b2	CAC1	CAC3	293	294
AVDVLQQMPLKEK-YQKSTELLIR-a11-b3	CAC1	H3	328	57
AVDVLQQMPLKEK-RYQKSTELLIR-a11-b4	CAC1	H3	328	57
AVDVLQQMPLKEK-LFTAGGDNKVR-a11-b9	CAC1	CAC2	328	38
EKTDDELQSLLAQVPHK-YQKSTELLIR-a2-b3	CAC1	H3	330	57
EKTDDELQSLLAQVPHK-RYQKSTELLIR-a2-b4	CAC1	H3	330	57
EKTDDELQSLLAQVPHK-NTKLLYDYLNTNSTK-a2-b3	CAC1	CAC3	330	31
KFVGPLIPTICLK-YQKSTELLIR-a1-b3	CAC1	H3	444	57
RKFVGPLIPTICLK-KLPFQR-a2-b1	CAC1	H3	444	65
KFVGPLIPTICLK-KLPFQR-a1-b1	CAC1	H3	444	65
RKFVGPLIPTICLK-YQKSTELLIR-a2-b3	CAC1	H3	444	57
AEVIIETDGPIDPFKEPK-SVLKLPYK-a15-b4	CAC1	CAC2	489	339
AEVIIETDGPIDPFKEPKTSSLPSK-SVLKLPYK-a15-b4	CAC1	CAC2	489	339
AEVIIETDGPIDPFKEPK-EAGGIVNMLPVKK-a15-b12	CAC1	CAC2	489	447
AEVIIETDGPIDPFKEPK-KIPCNSSDSKK-a15-b10	CAC1	CAC2	489	457
SNSDLQAQTASQSQSPEKK-IAKAELPCPGDVLR-a18-b3	CAC1	CAC2	518	235
LFDGVQDSTFSLGTVTEIAQKNLPQYNK-IAKAELPCPGDVLR-a21-b3	CAC1	CAC2	553	235
LFDGVQDSTFSLGTVTEIAQKNLPQYNK-EAGGIVNMLPVKK-a21-b12	CAC1	CAC2	553	447

LFTAGGDNKVRIWK-SRSSSPCSKR-a9-b9	CAC2	CAC1	38	129
KIESLDFLGSLTHHEQAINVIR-AVDVLQQMPLKEK-a1-b11	CAC2	CAC1	57	328
IAKAELPCPGDVLR-SSGKGDLPR-a3-b4	CAC2	CAC1	235	577
IAKAELPCPGDVLR-NTIKEYAIR-a3-b4	CAC2	CAC1	235	568
IAKAELPCPGDVLR-QKAMITDPMDLLR-a3-b2	CAC2	CAC1	235	521
IAKAELPCPGDVLR-DAVTYTEHAKRK-a3-b10	CAC2	H4	235	78
SGILNSAGGVKNRPAIR-SSGKGDLPR-a11-b4	CAC2	CAC1	306	577
SGILNSAGGVKNRPAIR-NTIKEYAIR-a11-b4	CAC2	CAC1	306	568
KPALMAAFSPVFYETCQK-KFVGPLIPTICLK-a1-b1	CAC2	CAC1	318	444
KPALMAAFSPVFYETCQK-RKFVGPLIPTICLK-a1-b2	CAC2	CAC1	318	444
KPALMAAFSPVFYETCQKSVLK-SSGKGDLPR-a18-b4	CAC2	CAC1	335	577
KPALMAAFSPVFYETCQKSVLK-TSSLPSKR-a18-b7	CAC2	CAC1	335	499
KPALMAAFSPVFYETCQKSVLK-QKAMITDPMDLLR-a18-b2	CAC2	CAC1	335	521
IEPPAMHAEPLDTDESAVAAKNQR-SSGKGDLPR-a21-b4	CAC2	CAC1	432	577
ISTLGHIKWSSLNNFDMDEMEFKPENSTR-YQKSTELLIR-a8-b3	CAC3	H3	92	57
QSKISHPFETK-IGNFFKK-a3-b6	CAC3	CAC1	171	235
IRQSKISHPFETK-KLSDSNTPVVEK-a5-b1	CAC3	CAC1	171	236
QSKISHPFETK-LKKEEEIR-a3-b3	CAC3	CAC1	171	212
QSKISHPFETK-LKEEAK-a3-b2	CAC3	CAC1	171	219
QSKISHPFETK-LKKEEEIR-a3-b2	CAC3	CAC1	171	211
QSKISHPFETK-RKEEER-a3-b2	CAC3	CAC1	171	205
EKHDGGVNSCR-ELKVEEER-a2-b3	CAC3	CAC1	294	172
EKHDGGVNSCR-ELLRQEEKK-a2-b8	CAC3	CAC1	294	166
FNYKNSLILASADSNGR-RKIDDELLNSK-a4-b2	CAC3	CAC1	307	282
FNYKNSLILASADSNGR-KIDDELLNSK-a4-b1	CAC3	CAC1	307	282
KLPFQRLVR-QEEKKK-a1-b4	H3	CAC1	65	166
DNIQGITKPAIR-ELKVEEER-a8-b3	H4	CAC1	32	172
GLGKGGAK-HRKELLR-a4-b3	H4	CAC1	13	158

DNIQGITKPAIRR-EKELKK-a8-b2	H4	CAC1	32	144
DAVTYTEHAKR-EIAQDFKTDLR-a10-b7	H4	H3	78	80

Supplementary Table 3. Cross-linked peptides of yeast CAF-1 _HistoneH3/H4_Asf1

Cross-linked Peptide ID	Protein1	Protein2	Position1	Position2
FLTKEKDVITLDDPK-MSGRGKGGK-a6-b6	CAC1	H4	39	6
LKKEEEIR-EKLQSNR-a2-b2	CAC1	CAC3	211	287
KLSDSNTPVVEK-EKLQSNR-a1-b2	CAC1	CAC3	236	287
KLSDSNTPVVEK-QSKISHPFETK-a1-b3	CAC1	CAC3	236	171
SDYEKFFLPFYAK-TKHGSTR-a5-b2	CAC1	CAC3	252	161
SDYEKFFLPFYAK-QSKISHPFETK-a5-b3	CAC1	CAC3	252	171
KIDDELLNSKDK-EKLQSNR-a10-b2	CAC1	CAC3	291	287
IDDELLNSKDK-EKLQSNR-a9-b2	CAC1	CAC3	291	287
DKTSSDDLLNWLQSR-EKHDGGVNSCR-a2-b2	CAC1	CAC3	293	294
RKAVDVLQQMPLK-LFTAGGDNKVR-a2-b9	CAC1	CAC2	317	38
EKTDDELQSLLAQVPHK-NTKLLYDYLNTNSTK-a2-b3	CAC1	CAC3	330	31
EKTDDELQSLLAQVPHK-EKHDGGVNSCR-a2-b2	CAC1	CAC3	330	294
KFVGPLIPTICLK-KLPFQR-a1-b1	CAC1	H3	444	65
KFVGPLIPTICLK-YQKSTELLIR-a1-b3	CAC1	H3	444	57
AEVIIETDGPIDPFKEPK-SVLKLPYK-a15-b4	CAC1	CAC2	489	339
AEVIIETDGPIDPFKEPK-SGILNSAGGVKNRPAIR-a15-b11	CAC1	CAC2	489	306
AEVIIETDGPIDPFKEPKTSSLPSK-SVLKLPYK-a18-b4	CAC1	CAC2	492	339
RSNSDLQAQTASQSQSPEKK-IAKAELPCPGDVLR-a19-b3	CAC1	CAC2	518	235
LFTAGGDNKVR-YQKSTELLIR-a9-b3	CAC2	H3	38	57
IAKAELPCPGDVLR-NTIKEYAIR-a3-b4	CAC2	CAC1	235	568
IAKAELPCPGDVLR-SSGKGDLPR-a3-b4	CAC2	CAC1	235	577
IAKAELPCPGDVLR-DAVTYTEHAKR-a3-b10	CAC2	H4	235	78
IAKAELPCPGDVLR-QKAMITDPMDLLR-a3-b2	CAC2	CAC1	235	521
KPALMAAFSPVFYETCQK-KFVGPLIPTICLK-a1-b1	CAC2	CAC1	318	444

KPALMAAFSPVFYETCQK-RKFVGPLIPTICLK-a1-b2	CAC2	CAC1	318	444
KPALMAAFSPVFYETCQKSVLK-KEEEIRLK-a18-b1	CAC2	CAC1	335	212
KPALMAAFSPVFYETCQKSVLK-TSSLPSKR-a18-b7	CAC2	CAC1	335	499
KPALMAAFSPVFYETCQKSVLK-QTIKNTIK-a18-b4	CAC2	CAC1	335	564
IEPPAMHAEPLDTDESAVAAKNQR-AEVIIETDGPIDPFKEPK-a21-b15	CAC2	CAC1	432	489
NTKLLYDYLNTNSTK-AVDVLQQMPLKEK-a3-b11	CAC3	CAC1	31	328
QSKISHPFETK-LKEEAKER-a3-b6	CAC3	CAC1	171	223
QSKISHPFETK-LKEEAK-a3-b2	CAC3	CAC1	171	219
QSKISHPFETK-LKKEEEIR-a3-b3	CAC3	CAC1	171	212
QSKISHPFETK-RAELKK-a3-b5	CAC3	CAC1	171	184
FNYKNSLILASADSNGR-RKIDDELLNSK-a4-b2	CAC3	CAC1	307	282
FNYKNSLILASADSNGR-KIDDELLNSK-a4-b1	CAC3	CAC1	307	282
KLPFQRLVR-QEEKKK-a1-b4	H3	CAC1	65	166
KLPFQRLVR-QEEKKK-a1-b5	H3	CAC1	65	167
